# Impact of endoscopic laser cricopharyngeal myotomy on lower oesophageal sphincter physiology

**DOI:** 10.1308/rcsann.2022.0119

**Published:** 2023-01-23

**Authors:** S Perring, SAR Nouraei

**Affiliations:** ^1^ University Hospitals Dorset NHS Foundation Trust, UK; ^2^ University of Southampton, UK; ^3^ Nottingham University Hospitals NHS Trust, UK

**Keywords:** Cricopharyngeus, Laser myotomy, Oesophageal physiology

## Abstract

Dysphagia is a watershed symptom that localises poorly. Dividing causes of dysphagia into oropharyngeal and oesophageal does not always best serve the patient. We report the case of a patient whose diagnosis and treatment required three separate specialist referrals to two specialties, with 18 months passing between initial referral and definitive treatment. The predominant pathology was isolated upper oesophageal sphincter dysfunction that responded well to laser cricopharyngeal myotomy. Following surgery, patient symptoms resolved and lost weight was regained. High-resolution manometry showed that the upper sphincter resting pressure had fallen from 117±45 to 21±11mmHg, but the lower sphincter resting pressure had risen, albeit without symptoms, from 16±8 to 44±17mmHg (*p<*0.001 in both cases). Surgery on upper oesophageal sphincter in the presence of lower oesophageal sphincter incompetence is known to lead to intractable regurgitation and pneumonia, and this novel physiological observation further emphasises the need to holistically consider the patient and to systematically evaluate the entire swallowing system before undertaking invasive interventions.

## Background

Swallowing disorders have historically been divided into those that affect bolus transport between the oral cavity and the cervical oesophagus, with airway protection, and those that affect bolus transport through the oesophagus and into the stomach, with regurgitation prevention.^1^ Separating the many causes of dysphagia into oropharyngeal and oesophageal in this way provides an empirical framework for organising patient referral pathways. It does not, however, encourage a holistic approach to evaluating a symptom that can be caused by different pathologies in different parts of the brain and multiple tissues and structures between the lips and the stomach.^1^

Dysphagia due to oesophageal sphincter dysfunctions presents specific challenges in terms of identifying the most significant aetiology, and minimising life-threatening treatment-related complications and mortality. Here we describe a novel physiological reaction to surgical division of the upper oesophageal sphincter that further highlights the need for meticulous patient selection.

## Case history

A 63-year-old man with no significant morbidities was referred to the gastrointestinal suspected cancer pathway following 3 months of persistent pharynx-localised dysphagia. He was discharged after a gastroscopy that showed a small hiatus hernia and a non-obstructive Schatzki’s ring.

**Figure 1:**
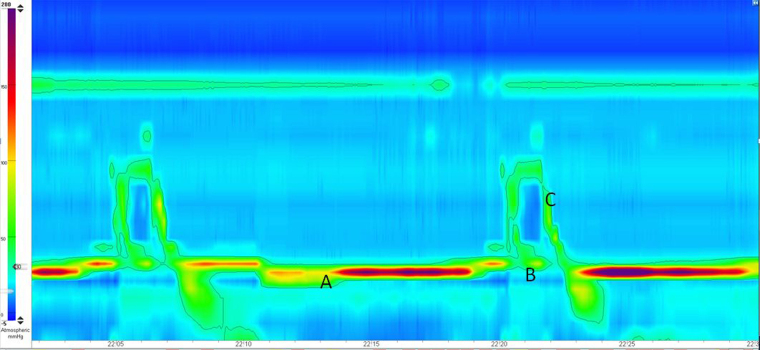
Clouse plot of the perioperative high-resolution manometry. Measurements were made using an MMS solar water-perfused system and a 24-channel disposable manometry catheter. This plot is of the pharynx and the upper oesophageal sphincter during wet swallowing: (A) hypertensive upper oesophageal sphincter, (B) poor upper sphincter relaxation and (C) weak and poorly coordinated pharyngeal peristalsis. The timescale is given in seconds.

He was concurrently re-referred to gastroenterology and otolaryngology 11 months later, after remaining symptomatic and losing 10kg in weight. Gastroenterology organised a barium swallow that showed cricopharyngeal hypertrophy and no pharyngeal pooling or aspiration. Otolaryngology organised oesophageal physiology and a Transnasal Panendoscopy. Endoscopy showed no major mucosal pathologies and oesophageal physiology confirmed upper sphincter hypertension with distorted pharyngeal swallow (Figure 1) but no evidence of oesophageal motor disorders: distal contractile integral was 1312mmHg/cm/s and integrated relaxation pressure was 4mmHg. Resting lower oesophageal sphincter pressure was 16mmHg, and proximal and distal acid exposure times were 0% and 0.5% on 24h pH impedance manometry, respectively. There were only 18 acid or non-acid impedance events and Mean Nocturnal Baseline Impedance was >3,000Ω. There was no nasal regurgitation, dysarthria, gait disorders, choreiform movements, eyelid droop or focal neurology to point towards a neurological cause.

**Figure 2:**
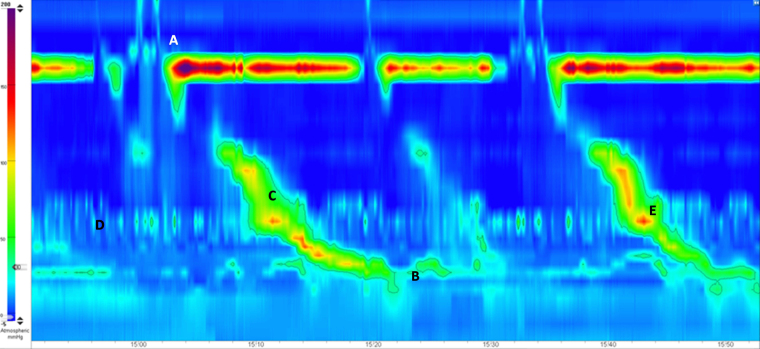
Clouse plot of the oesophagus: (A) hypertensive upper oesophageal sphincter, (B) normal lower oesophageal sphincter, (C) adequate peristalsis, (D) extrinsic oesophageal pressurisation, and (E) extrinsic oesophageal pressurisation adversely interfering with free progression of the peristaltic wave. The timescale is given in seconds.

High-resolution manometry raised the possibility of extrinsic intrathoracic oesophageal compression (Figure 2). Computed tomography scan identified non-specific mediastinal lymphadenopathy and there was no evidence of systemic diseases as part of evaluating this finding. The respiratory team provided reassurance following a positron emission tomography scan and a lung and multidisciplinary team discussion. Invasive evaluations such as endobronchial ultrasound were not deemed necessary.

The patient then underwent a panendoscopy and pharyngo-oesophageal dilation with short-lived improvement in dysphagia. He did not develop regurgitation or pneumonia, but continued to have dysphagia and was unable to regain weight.

**Figure 3:**
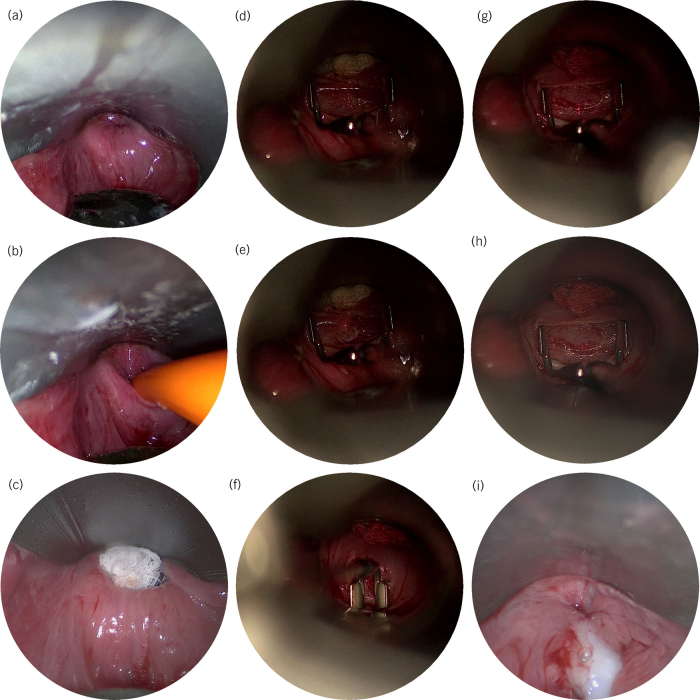
Endoscopic laser cricopharyngeal myotomy. (a) Access to the pharyngo-oesophageal junction is achieved with a Weerda distending pharyngoscope (Karl Storz GmBH, Tuttlingen, Germany). (b) The oesophageal lumen is intubated with a slim Boujie and if necessary, visualised using an ultrathin oesophagoscope. (c) A wet mastoid swab is placed within the oesophageal lumen to provide tissue traction and to protect distal structures. (d–h) A linear incision is made with CO_2_ laser, which is deployed using the Lumenis Digital AcuBlade® system (Lumenis Inc., San Jose, US) with a 2mm straight line geometry and at 8W super-pulse settings. The CO_2_ laser has a low tissue penetration depth and provides significant safety advantages over diffuse energy sources like diathermy and coblation and especially in terms of reducing injury to the buccopharyngeal fascia. A Lindholm vocal cord retractor (Karl Storz) is used to demonstrate muscle fibres and apply tension. The myotomy begins superiorly (closest to the oesophageal opening) and great care is taken not to violate the buccopharyngeal fascia (the floor of the dissection is best seen in panel H). Furthermore, muscle is divided in the midline where the buccopharyngeal membrane and the alar fascia fuse into a thicker fascial layer. The fascial layer that forms the safe limit of this dissection is the same fascial layer that forms the limit of dissection during robotic or laser lateral oropharyngectomy (i) After the myotomy is completed, fibrin sealant (TISSEEL, Baxter International, Deerfield, US) is applied and the wound is closed with interrupted 4/0 Vicryl sutures (Ethicon Inc., Raritan, US).

Given the persistence of symptoms with temporary benefit from dilation, given absence of oesophageal motor disorders or systemic diseases like scleroderma causing dysmotility, given absence of clinical evidence for neurological upper sphincter dysfunction that would have made us consider a trial of botulinum toxin injection, and given absence of pathological reflux, lower sphincter incompetence, or post-dilation regurgitation, the patient underwent an endoscopic laser cricopharyngeal myotomy (Figure 3) 18 months after the initial referral. He experienced one episode of chest pain on the second postoperative day that was investigated with a coronary angiogram and cardiac magnetic resonance imaging, which showed no evidence of acute coronary syndrome or cardiac injury. He otherwise had an uneventful recovery.

**Figure 4:**
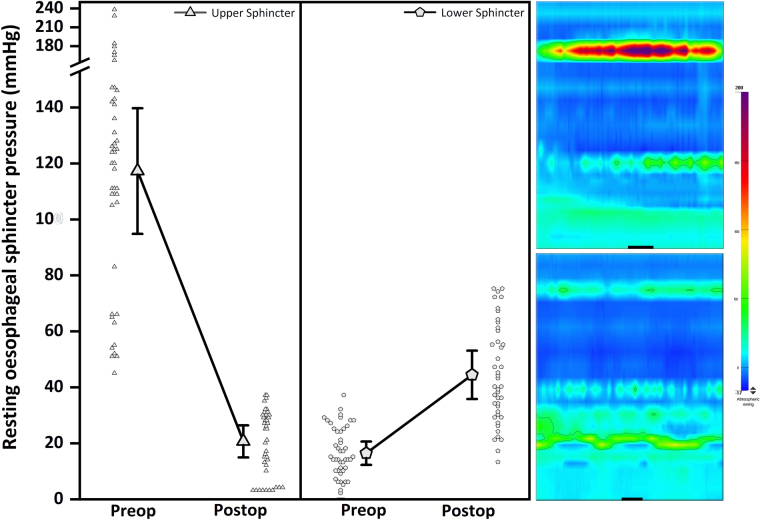
Representative preoperative and postoperative oesophageal Clouse plots. Multiple pressure measurements at 0.2s intervals over at least two separate representative periods show significant changes in upper and lower oesophageal sphincter pressures (*p<*0.001) following laser cricopharyngeal myotomy. The time scale (black line) on Clouse plots represent 1second.

Four months after surgery, the patient’s dysphagia had remained resolved, lost weight had been regained, and the patient was able to resume heavy physical work. Postoperative manometry showed normalisation of upper sphincter pressure, no change in oesophageal motor function, and a significant but asymptomatic rise in the resting lower sphincter pressure (Figure 4). The patient gave written informed consent for the publication of this case.

## Discussion

This case demonstrates challenges in expeditiously diagnosing and managing dysphagia, which is a watershed symptom. It took three separate referrals to make a diagnosis and over that time, the patient decompensated physically and became less economically active. Swallowing is one of Foregut’s most complex functions and the time-honoured but time-inefficient fragmentation of its evaluation and treatment^1^ does not put the patient at the centre of care. Moreover, although this patient had a benign diagnosis, >5% of cancer patients receive an ‘interspecialty referral’, which significantly reduces their survival.^2^ We believe that patient-centred integration of dysphagia services are long overdue.

Physiologically, the postoperative fall in upper sphincter pressure was accompanied by a rise in lower sphincter pressure. This novel observation is readily explicable by considering the pharynx, larynx and the oesophagus and their sphincters as the single functional unit that they are, that coordinate deglutition in one direction, belching and vomiting in the other direction, and maintain anti-reflux and airway-protective barriers.

The upper and lower oesophageal sphincters are comprised of striated and smooth muscle, respectively, and could be individually affected by different disease processes. Examples include this patient and inclusion-body myositis^3^ for upper sphincter dysfunction, and isolated oesophagogastric outflow obstruction for lower sphincter dysfunction. As this case demonstrates, however, modifying oesophageal sphincter function even in an isolated dysfunction causes compensatory responses within the unaffected sphincter. Failing to consider the baseline function of the ‘unaffected’ sphincter and the ‘physiological reserve’ within the whole system can cause major complications^3^ and lead to avoidable harm, especially when symptoms of pharyngeal outflow obstruction arise not from isolated upper sphincter dysfunction, but from wider imbalances between bolus admittance and regurgitation-prevention functions. These wider imbalances can arise from gastro-oesophageal and intra-oesophageal reflux due to lower oesophageal sphincter incompetence and oesophageal motor disorders, respectively.

Treating suspected upper sphincter dysfunction thus requires a physiological understanding of the presenting symptom. Attempts at reducing pharyngeal outflow obstruction without assessing the risk of ‘cup-and-spill’ aspiration due to pharyngeal residue on the one hand,^4^ and aspiration due to intra-oesophageal or gastro-oesophageal reflux and regurgitation through an upper sphincter rendered incompetent on the other hand,^3^ expose the patient to significant risk of avoidable harm.^5^ In our practice therefore, all patients with suspected upper sphincter dysfunction must undergo fluoroscopic, manometric, and pH studies, to evaluate and balance potential benefits of improving dysphagia and reducing the risk of aspiration from post-swallow pharyngeal residue, against the risk of aspiration from intractable post-treatment intra-oesophageal and gastro-oesophageal regurgitation.

## Conclusions

This case demonstrates challenges in expeditiously managing dysphagia within the current fragmented patient referral pathways. It also provides physiological evidence of the intimate relationship between the upper and lower oesophageal sphincters and with that, the need to holistically characterise pharyngo-oesophageal function before undertaking treatment.
